# Plenary Abstracts

**DOI:** 10.1080/24740527.2022.2088024

**Published:** 2022-08-12

**Pain and the developing nervous system – It all starts here**

Maria Fitzgerald

Department of Neuroscience, Physiology & Pharmacology, University College London, UK

This presentation will focus upon our understanding of how pain is processed in the developing spinal cord and brain. The neurobiological, mechanistic approach provides (i) predictive and testable theories of early life pain from age-appropriate animal models using new technologies and (ii) a scientific framework for better measurement & treatment of pain in infants and children. Pain is learned in infancy – and so discoveries in this area are relevant to us all.

Newborn mammals display robust responses to noxious or tissue- damaging stimulation. These nociceptive or “pain” responses arise from neural activity at different levels of the central nervous system. Protective reflex movements and physiological reactions mediated by spinal cord and brainstem circuits are essential for the preservation of life and well- being but should not be equated with pain awareness. The unique sensation of pain and its unpleasant, threatening quality requires activity in the cortical and subcortical regions of the brain.

Here I will present our recent research on the maturation of pain processing in the young mammalian brain, drawing on data from human infants and laboratory rodent pups, and highlighting fundamental differences from pain processing in the adult. I will further show new evidence that early life pain alters adult brain functional connectivity, both within and between cortical areas involved in sensory and affective dimensions of pain. This data provides direct evidence that the cortical pathways underlying adult pain experience are shaped by events in infancy. Such discoveries are relevant to us all.

**Learning Objectives**: At the end of this session, participants will be able to:

- discuss recent advances in our knowledge of the developing nociceptive system;
- identify how a developmental neuroscience approach to pain behaviour and pain perception can help young people with pain; and
- explain how pain circuits in the adult brain are altered by early life pain experience.

**Treatment of neuropathic pain: Led and misled by clinical trials**

Nanna Brix Finnerup

Danish Pain Research Center, Department of Clinical Medicine, Aarhus University, Denmark

Neuropathic pain is a complex disabling neurological disorder caused by, for example, stroke, spinal cord injury, neuropathy and nerve injury, and is a growing global challenge. In this presentation, I will give an overview of the treatment options for neuropathic pain. I will present the NeuPSIG (Neuropathic Pain Special Interest Group of IASP) recommendations for the pharmacological treatment of neuropathic pain and new drug trials. Surprisingly few new treatments have been introduced and proven effective and safe for neuropathic pain over the past 120 years, and the drugs available have not been developed through bottom-up translational approaches but rather through empirical clinical observations. Main barriers for the failure to introduce new analgesics may include complexity and diversity of underlying pathophysiological mechanisms, poor animal-to-human translatability, heterogeneity of clinical pain phenotypes and mechanisms, and complexity of clinical trials with high placebo responses and low assay sensitivity. There is some evidence from clinical trials that patient profiling might be informative for deciding on certain treatments and I will discuss the advances in phenotype and mechanism-based treatment trials.

**Learning objectives**: At the end of the session, the participant will be able to:

- describe evidence-based pharmacological treatments of neuropathic pain;
- evaluate limitations of clinical trials; and
- exemplify phenotype-based treatment trials.

**Imaging pain processing in the brain and spinal cord of the awake, behaving mouse**

Allan Basbaum

Department of Anatomy, University California San Francisco, San Francisco, California, USA

Prior to the development of very rapidly acting general anesthetics, distinct stages of general anesthesia were readily recognized. For example, using ether, patients initially are unresponsive to normally pain-provoking stimuli. This Stage 1 is followed by an amnestic state and only after that is unconsciousness provoked. These interesting properties suggested to our laboratory that by monitoring the activity of different general anesthetics it might be possible to identify populations of neurons that are critical to the experience of pain. To this end we have used calcium imaging in the mouse to study the effects of isoflurane and nitrous oxide on the activity of neurons in the anterior cingulate cortex, a region generally associated with the affective component of the pain experience. It is of particular interest that nitrous oxide, in contrast to isoflurane has analgesic properties. In this presentation we will demonstrate unexpected differential effects of these two anesthetics on the activity of ACC neurons. In contrast to cortical studies of pain processing, spinal cord analyses have relied on recordings in anesthetized or semi-intact preparations. The ability to image activity of the same population of spinal cord neurons, long-term, in an awake preparation, we predict will provide important insights into mechanisms that underlie the transition from acute to chronic pain. Success in the endeavor required that optical imaging be sustained after laminectomy, a hurdle that we only recently overcame.

**Learning Objectives**: At the end of this session, participants will be able to:

- illustrate the advantages offered by calcium imaging to monitor CNS circuitry in behaving animals;
- describe the difference in cortical regulation provoked by different volatile general anesthetics and how this relates to their analgesic action; and
- illustrate the possibilities offered by imaging.

**Paradigmatic shifts in health research and the problem of pain in people with dementia**

Thomas Hadjistavropoulos

Department of Psychology and Centre on Aging and Health, University of Regina, Regina, Saskatchewan, Canada

Over the last 30 years there have been significant changes in the manner in which health research is conducted. Paradigmatic shifts increasingly have emphasized the importance of interdisciplinarity, knowledge translation, knowledge mobilisation, and partnerships with stakeholders. These paradigmatic shifts have moved us from fragmented unidisciplinary approaches to more integrated knowledge acquisition with a greater focus on application. Using a program of research on pain in dementia as an example, I will illustrate ways in which tackling complex, real world problems can lead traditionally-trained clinical health scientists to areas of scholarly inquiry that were previously foreign to them. Effectively addressing the problem of pain in dementia requires a combination of basic and clinical science, public policy, knowledge translation, cost investigations, implementation science, engineering, computer science and patient/caregiver partners.

**Learning objectives**: At the end of the session, the participant will be able to:

- summarize recent shifts in stakeholder/knowledge user expectations that have changed the way in which health research is conducted; and
- describe recent developments in pain assessment in dementia, associated challenges with implementation of research findings, and researcher adaptations to a new research environment that benefits from knowledge user input, implementation science methodologies and interdisciplinarity.

**Igniting the spark: How people with lived experience have transformed my career**

Katie Birnie

Department of Anesthesiology, Perioperative and Pain Medicine, and Department of Community Health Sciences, University of Calgary, Calgary, Alberta, Canada

**Learning objectives**: At the end of the session, the participant will be able to:

- demonstrate how people with lived experience have shaped and expanded my early career in pain research, care, and policy;
- illustrate historical and current examples of how people with lived experience have been critical to advancing pediatric pain research, care, and policy; and
- understand how partnership with people with lived experience contributes to more equitable pediatric pain management.

